# Harbour Porpoise Abundance in Portugal over a 5-Year Period and Estimates of Potential Distribution

**DOI:** 10.3390/ani12151935

**Published:** 2022-07-29

**Authors:** Andreia Torres-Pereira, Hélder Araújo, Fábio L. Matos, Jorge Bastos-Santos, Sara Sá, Marisa Ferreira, José Martínez-Cedeira, Alfredo López, Marina Sequeira, José Vingada, Catarina Eira

**Affiliations:** 1 Department of Biology & CESAM, Universidade de Aveiro, 3810-193 Aveiro, Portugal; fmatos@ua.pt (F.L.M.); saramsa@ua.pt (S.S.); a.lopez@ua.pt (A.L.); catarina.eira@ua.pt (C.E.); 2 ECOMARE, Universidade de Aveiro, 3810-193 Aveiro, Portugal; helder.araujo@socpvs.org (H.A.); mctferreira@socpvs.org (M.F.); spvs@socpvs.org (J.V.); 3 Portuguese Wildlife Society (SPVS), Estação de Campo de Quiaios, 3081-101 Figueira da Foz, Portugal; jorgeb.santos@gmail.com; 4 Coordinadora para o Estudio dos Mamíferos Mariños (CEMMA), Apdo, 156-36380 Gondomar, Spain; jmcedeira@yahoo.es; 5 Instituto da Conservação da Natureza e Florestas (ICNF), Av. da República 16, 1050-191 Lisboa, Portugal; marina.sequeira@icnf.pt

**Keywords:** Iberian harbour porpoise, aerial surveys, abundance and density estimates, habitat suitability, environmental variables, conservation, mitigation measures

## Abstract

**Simple Summary:**

The Iberian porpoise inhabits the Atlantic coast of Portugal and Spain. The population is relatively small and there is a high number of individuals accidentally captured in fisheries. Using airplane surveys, we estimated that there were only 2254 porpoises overall between 2011 and 2015 in the coast of Portugal. The highest annual number of porpoises was recorded in 2013 (3207 individuals). However, in the following year, our study revealed that the population had been reduced approximately by half (when an increase in stranded individuals was also registered). The northern area of Portugal presented the most suitable habitat for the Iberian harbour porpoise, where coastal fisheries represent a particularly important socio-economic activity. Measures to decrease fishing effort are urgently needed as well as detailed information on seasonal Iberian harbour porpoises’ use of space.

**Abstract:**

The Iberian porpoise population is small and under potentially unsustainable removal by fisheries bycatch. Recently, a marine Site of Community Importance (SCI) was legally approved in Portugal, but no measures ensued to promote porpoise conservation. Information about porpoise abundance and distribution is fundamental to guide any future conservation measures. Annual aerial surveys conducted between 2011 and 2015 show a low overall porpoise abundance and density (2254 individuals; 0.090 ind/km^2^, CV = 21.99%) in the Portuguese coast. The highest annual porpoise estimates were registered in 2013 (3207 individuals, 0.128 ind/km^2^), followed by a sharp decrease in 2014 (1653 individuals, 0.066 ind/km^2^). The porpoise density and abundance estimated in 2015 remained lower than the 2013 estimates. A potential distribution analysis of the Iberian porpoise population was performed using ensembles of small models (ESMs) with MaxEnt and showed that the overall habitat suitability is particularly high in the Portuguese northern area. The analysis also suggested a different pattern in porpoise potential distribution across the study period. These results emphasize the importance of further porpoise population assessments to fully understand the spatial and temporal porpoise habitat use in the Iberian Peninsula as well as the urgent need for on-site threat mitigation measures.

## 1. Introduction

A sharp decline in genetic diversity detected over the past 25 years indicates that the harbour porpoise population in the western Iberian Peninsula may be small and isolated [1]. Porpoises inhabiting upwelling areas in the western Iberian Peninsula and Mauritania are genetically differentiated from other populations [2,3,4] and a possible subspecies was proposed (*Phocoena phocoena meridionalis*) [3]. The harbour porpoises inhabiting Portuguese and Spanish waters (Iberian Peninsula) are considered a source population with individuals migrating to (but not from) northern and southern neighboring populations [3], and a hybridization zone for Iberian and north Atlantic porpoises was identified in the northern part of the Bay of Biscay [3,5]. However, migration from a southern population into the Iberian Peninsula was recently detected through molecular analysis of samples from individuals stranded in the Portuguese south coast [1].

Efforts to address the need for conservation measures applied to the harbour porpoise population in Portugal were initiated in 2010, including annual porpoise population surveys and trials of mitigation measures to reduce bycatch in fisheries [6,7]. These efforts culminated in the legal approval of an exclusively marine Site of Community Importance (SCI) later in 2019 (PTCON0063, RCM17/2019), four years after the deadline imposed by the European Commission for Portugal to define an SCI for porpoises, as required by the European Habitats Directive for Annex II species. Despite the pressure for site designation in Portugal (which should have framed the application of a conservation plan), there have been no further measures effectively applied to promote the conservation of porpoises, which should have been focused on bycatch mitigation, to address the most important mortality source [7]. Similar situations have been described in other EU countries with respect to the conservation of transnational species [8].

The porpoise population in Portugal is, in fact, known to be particularly susceptible to interactions with coastal fishing gears [6]. The different Portuguese fishing fleets (5257 registered boats in 2019 operating off the continental coast, [9]) include a large portion of vessels operating in coastal areas. Preliminary results on porpoise bycatch emphasized alarming rates of mortality, particularly in gill nets and trammel nets, and in beach-seines [7]. Presently, mortality is monitored through the national strandings network, and data supports that fisheries bycatch continues to be a major issue to porpoise population persistence and abundance. Information about abundance and distribution of threatened species such as the Iberian harbour porpoise are of fundamental importance for dealing with such conservation issues [10,11]. In particular, abundance estimates and by-catch mortality assessments should be used to guide management actions to prevent unsustainable by-catch levels [12].

In Portuguese coastal waters, we conducted a standardized aerial survey protocol replicated annually over a 5-year period in order to collect harbour porpoise sightings data. Aerial surveys are considered a standardized and cost-efficient census methodology providing robust estimates [13], constituting the principal technique used in several monitoring programs [11,14,15]. We then used Ensembles of Small Models (ESMs) based on ecological niche modelling to estimate the potential distribution of harbour porpoises in the western Iberian Peninsula. ESMs have been successfully applied to model rare species [16] while ensemble approaches consistently show higher reliability over single models [16,17].

The aims of this study were to understand how harbour porpoises were distributed in Portugal within the study period, how abundance and densities evolved over time, and also to identify the most suitable areas for porpoises in the western Iberian Peninsula. Our findings on abundance and density estimates in Portugal are also discussed considering results from other surveys on Iberian waters (SCANS II and III).

## 2. Materials and Methods

### 2.1. Study Area

**Figure 1:**
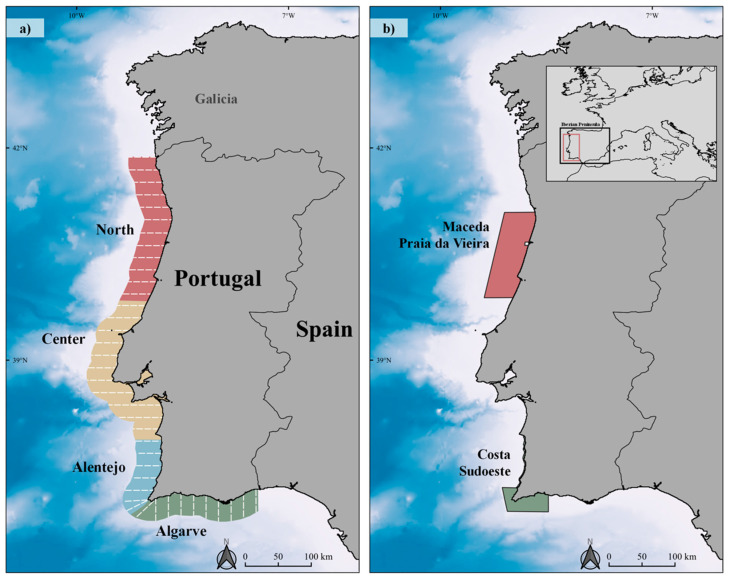
Study area in the continental coast of Portugal. (**a**) Overview of the survey area divided into four sectors: North, Centre, Alentejo, and Algarve; designed line transects (dashed white line); (**b**) Sites of Community Importance Maceda-Praia da Vieira (PTCON0063) and Costa Sudoeste (PTCON0012).

The Portuguese exclusive economic zone (EEZ) covers 327,667 km^2^, including a 23,728 km^2^ continental shelf, which is generally narrow although wider profiles occur associated to the northern coast of the country. The study area (25,052.52 km^2^) was defined by a 20-nautical-mile (nm) buffer along the Portuguese coastline (latitude ranging from 36.5° N to 41.9° N, Figure 1a) and the four sectors were separated according to oceanographic characteristics, but also to accommodate airplane logistic needs and proximity to airfields: North (6710.99 km^2^), Centre (9154.15 km^2^), Alentejo (3424.86 km^2^), and Algarve (5771.52 km^2^) see [18]). In the study area, there are two Sites of Community Importance (SCIs, NATURA 2000) (see Figure 1b) whose marine areas, representing 28.09% of the Portuguese continental shelf, are dedicated to the protection of cetaceans, particularly harbour porpoises and bottlenose dolphins [19,20].

### 2.2. Survey Design and Data Collection

This study was conducted in the framework of a broad-scale marine megafauna survey to obtain annual density and abundance estimates for several cetacean and seabird species. Annual aerial surveys were conducted in September between 2011 and 2015 except for 2013, when the survey was conducted in early October. High-wing airplanes equipped with two bubble windows (to allow scanning directly under the plane) were used to survey over a predesigned, systematic set of parallel 20-nm-long transect lines, which were obtained using DISTANCE [21] to ensure equal coverage probability (Figure 1a). Transects were separated by 10 nm and perpendicular to the coast, since transect direction should be parallel to any hypothetical gradients [22].

Flights were performed at an average speed of 100 knots (185 km/h), at an altitude of 500 ft (150.4 m). Surveys were conducted in weather conditions safe for flying operations (no fog or rain) with visibilities > 3 km and under Beaufort (windforce) scale ≤ 3, which are considered suitable for porpoise sightings [23,24]. Survey data was collected by two trained observers and one data recorder. The time and airplane positions were logged automatically every one second with a Garmin Global Positioning System (GPS).

Sighting data were registered in customized forms, including GPS position, declination angle (measured by a hand-held clinometer when an animal or group of animals was abeam), species identification, group size, presence of calves, behavior, swimming direction relative to the transect, and detection cue. For each sighting, the distance from the transect line to the animal or group of animals was calculated using the declination angle and the airplane’s altitude [13,23].

### 2.3. Abundance and Density Analysis

Conventional Distance Sampling (CDS) [22] was used to estimate abundance and density of the harbour porpoise in Portuguese continental waters. All analyses were carried out in Distance 7.3 Release 2 [21]. Distance sampling is a widely used technique in which distances from a transect line to the detected objected are modelled to obtain accurate estimates of the density and/or abundance of biological populations [21,22]. Distance sampling considers several assumptions for the collected data, namely (1) all objects on the line are detected (detection probability g(0) = 1), (2) detections are made at their initial locations, (3) the distance measurements are exact, (4) the group sizes are recorded without error, and (5) sampled transects are representative of the entire survey region [22]. In the present study, we assumed that the detection probability falls off smoothly from 1 as a function of distance from the track line. This function is known as the detection function [22]. However, the assumption that all animals on the line are detected may be violated because observers may, in fact, fail to detect some of the animals. In order to minimize this possible underestimation, different g(0) values for the harbour porpoise are available from estimates performed in SCANS II and SCANS III airplane surveys for moderate or good observation conditions, respectively: g(0) = 0.31 or 0.45 [11] and g(0) = 0.279 or 0.364 [15]. In this study, because we did not have our own g(0) estimate, we used the SCANS III g(0) = 0.364 defined for good conditions, to match the survey conditions in our study. The same aircraft type (Partenavia P68) and observer team involved in the present study were later involved in the SCANS III survey, thus contributing to the g(0) estimates in SCANS III.

To analyze the data, we tested combinations of the most recommended models for detection functions (uniform key with cosine adjustments, half-normal key with cosine adjustments, half-normal key with Hermite polynomial adjustments, and hazard-rate key with simple polynomial adjustments) [21]. The Akaike’s Information Criterium (AIC) provides a relative measure of the best fit [21]; therefore, model selection was guided by the lower AIC value. The effect of group size on detection probability was tested by fitting a regression of group size log against the detection probability [21,22]. The mean group sizes obtained from the regression (expected group sizes) were used instead of the observed mean group size, if there was a significant effect at α=0.15. Annual and overall abundance and density estimates, as well as a global detection function, were calculated for the whole study area, using the data from the five sampling years. The coefficient of variation (CVs) and 95% confidence intervals (CIs) were calculated by bootstrapping (999 replicates) within strata, using transects as sampling units [22]. Additionally, abundance and density estimates (and respective CVs and 95% CIs) were obtained for each sector, considering the combined five-year dataset. We used the Kruskal-Wallis test and post-hoc Dunn’s test to compare median group sizes between sectors.

### 2.4. Ecological Niche Modelling (ENM)

We used ecological niche models (ENMs), which are statistical and machine-learning approaches to relate species distribution data (observations) and environmental predictor variables to describe, understand, and predict the distribution of the species, e.g., [25].

#### 2.4.1. Environmental Data

Several eco-geographical variables (EGVs) are known as potentially relevant habitat drivers for cetaceans. According to literature, we built the ESM using information on bathymetry, distance to coast [26,27,28], slope [26,29], sea surface temperature (SST), and chlorophyll-a concentration (Chl-a) [27,30,31]. We also tested the use of pH, salinity, silicate, dissolved oxygen, phosphate, and nitrate [31] as environmental predictors as well as the ammonium (NH_4_^+^) considering its importance for phytoplankton growth and marine trophic webs, e.g., [32]. All static and dynamic variables were extracted from online repositories and incorporated in a preliminary analysis (Table 1). For these variables, we considered the monthly average values of the month previous to the airplane surveys to account for a time lag between dynamic variables and their potential effect on higher trophic levels, e.g., [33,34,35].

The slope and distance to coast variables were computed from bathymetry using the built-in tools provided by the *raster* package for R [37]. The dynamic environmental data were obtained at a 1/12° horizontal resolution (approximately 8 km) from data assimilative numeric models available from the Copernicus Marine Environment Monitoring Service (CMEMS). The variables were further interpolated to match the bathymetric data resolution (1 km^2^) using the inverse distance weighting method, available in the R gstat package [38,39].

The correlation between EGVs was assessed (Figure S1) using the virtualspecies package for R [40]. Highly correlated variables (Pearson correlation > 0.7; [41,42]) such as distance to coast, pH, salinity, silicate, dissolved oxygen, phosphate, and nitrate were excluded from further analyses. The selection was made so that the set of selected variables remained constant across the study period. Therefore, the final set of EGVs included in the analysis were: bathymetry and slope (as static variables), and sea surface temperature (SST), chlorophyll-a (Chl-a) and ammonium concentration at the sea surface (as dynamic variables).

#### 2.4.2. Modelling

We adopted an ensemble approach of ecological niche models to reduce the uncertainty levels of models’ prediction and assessment of harbour porpoise habitat suitability in the present study [17]. We further used an ensemble of small models (ESMs) approach since this modelling technique represents a novel and robust strategy to model rare species and can incorporate several standard modelling algorithms [16,43]. Maximum entropy modelling (MaxEnt) was selected as the modelling algorithm, since it is considered very efficient with irregular data and small sample size [28,44,45] and when handling minimal location errors [46] and collinearity [47,48]. Considering the incomplete information about the distribution of the target species, MaxEnt estimates the probability distribution of maximum entropy (i.e., the most spread out or closest to uniform) given a set of environmental characteristics at known occurrence sites that represent the incomplete information about the distribution of the species [49]. To assess the habitat suitability for the Iberian Harbour porpoise population across the western Iberian coast, the model was extrapolated to unsampled areas, including Galician waters in northwestern Spain (extent area: from 36° N to 44° N and 11° W to 6° W).

The modelling procedure was conducted using R version 3.6.0 [50] and the ecospat version 3.1 [51] and biomod 2 version 3.4.6 [52] packages. For each year, bivariate models (i.e., models that contain only two EGVs at a time) were built using the subset of selected EGVs and considering every possible combination. For the model fitting and evaluation, 10 datasets were generated with 10,000 background data points. Presences and background data were equally weighted (i.e., prevalence equal to 0.5) in the calibration process of the models. The assessment of each bivariate model was performed with 10 runs using 80% of the total records for model training and 20% for testing. The bivariate models were then combined according to their performance into a final ensemble prediction [16]. Note that the bivariate models with a Somers’ D score lower than 0.5 (model performance worse than random prediction), were not considered for the ensemble model prediction. The final ensemble prediction resulted from the calculation of the weighted average of the selected bivariate model runs (weights defined according to the Somers’ D score of each bivariate run), thus allocating more weight to models with better performances [16]. This approach avoids model’s overfitting by keeping an appropriate ratio between the number of EGVs and the number of observations [43]. The ensemble model prediction can be interpreted as a habitat suitability index (HSI) ranging between 0 (low suitability) and 1 (high suitability). Finally, we computed the average of the five annual ensemble predictions to obtain a single estimation to identify areas that are more frequently classified with higher HSI. Additionally, we calculated the coefficient of variation (i.e., standard deviation/mean) of the probabilities using the annual models; the coefficient of variation can be interpreted as a measure of uncertainty where lower scores represent better model estimates.

The predictive power of the model was evaluated using the Continuous Boyce Index (CBI) [53]. The CBI ranges from −1 to 1, where 0 means the model does not differ from the random forecast, positive values indicate that areas with a high number of occurrences are scored with high suitability values (good model performance), while negative values indicate that areas with a high number of occurrences are scored with low suitability values (model performed poorly) [54]. The contribution of each EGV for the annual ensemble models was assessed by calculating the mean weight of all bivariate models including the variable of interest, rescaled by the variable with the highest weight to a range between 0 and 1 [55].

## 3. Results

### 3.1. Abundance and Density Analysis

**Figure 2:**
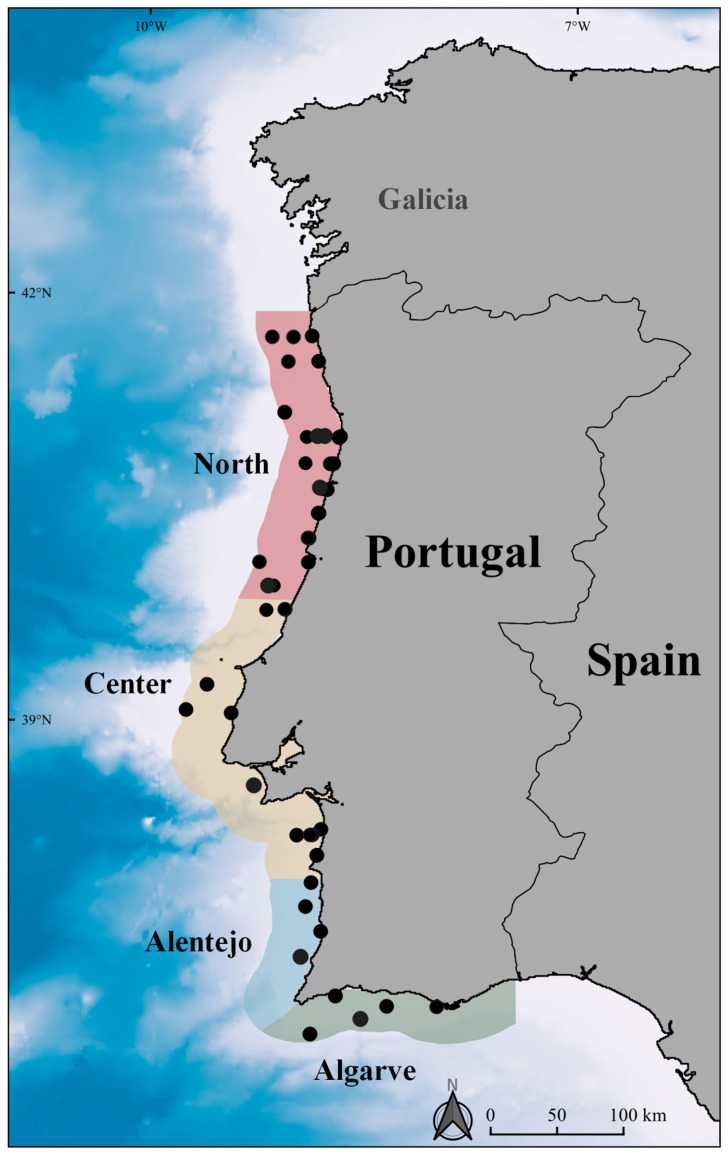
Harbour porpoise sightings during aerial surveys between 2011 and 2015 in the survey area divided into four sectors: North, Centre, Alentejo, and Algarve.

From 2011 to 2015, a total of 195 transects and a total distance of 8635 km were surveyed by airplane. Overall, 56 sightings were registered corresponding to 83 harbour porpoises (Figure 2, Table 2). The number of sightings varied between 6 in 2011 (10.7% of all sightings) and 16 (28.6% of all sightings) in 2012 and in 2013 (Table 2).

The total number of sightings was used in DISTANCE to estimate the detection function since truncation did not improve the detection function quality, despite the lower detection probability for distances over 200 m from the centre line.

**Figure 3:**
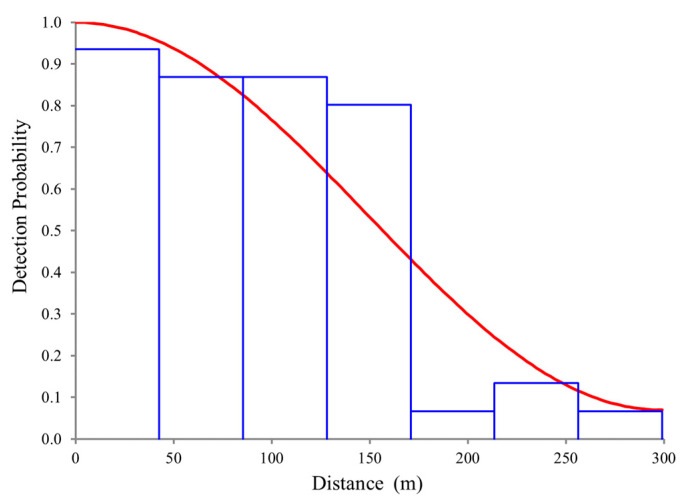
Histogram of harbour porpoise sightings (*n* = 56) in the survey area at given distances from the transect line and corresponding global detection function using the uniform key with cosine adjustments (red line).

We tested combinations of the most recommended models and due to lower AIC value (614.17), the uniform key with cosine adjustments was the best model combination using data from all sampled years, when comparing to models using the half normal key model with cosine adjustments (AIC = 614.73), half-normal key with hermite polynomial adjustments (AIC = 614.73) and the hazard-rate key with simple polynomial adjustments (AIC = 614.84). The histogram presenting the probability of detection function shows a general decreasing pattern with increasing distance from the transect line, and the resulting estimate of the effective strip width (ESW) was 159.90 m (95% CI: 143.66–177.98 m) (Figure 3).

Between 2011 and 2015, the overall estimated abundance (using g(0) = 0.364) was 2254 individuals (CV = 21.99%) with a density of 0.090 ind/km^2^ (Table 2). The lowest harbour porpoise abundance (1196 individuals) and density estimates (0.048 ind/km^2^) were registered in 2011, whereas the highest harbour porpoise estimated annual abundance and density (respectively, 3207 individuals and 0.128 ind/km^2^) were registered in 2013 followed by a sharp decrease in 2014, with only 1653 estimated individuals and a 0.066 ind/km^2^ density (CV = 43.27%). The overall expected group size was 1.39 individuals (CV = 7.80%). In all analysed data frames, the expected group size value was chosen over the observed value (to account for possible group size bias due to increasing distance from the transect line).

Considering the different sectors, the highest number of harbour porpoise sightings were registered in the North sector (*n* = 27), as well as the highest density estimates (0.140 ind/km^2^, CV = 43.42%). On the other hand, only four sightings were registered in the smaller Alentejo sector (Figure 2, Table 3). Although no significant differences were detected between years in the median number of individuals per group (Kruskal–Wallis test, H = 0.9763, *p* = 0.475), the median group size was higher in the Algarve sector (Kruskal–Wallis test, H = 8.69, *p* = 0.0337 and Dunn’s test *p* = 0.0301) when compared to the other sectors.

### 3.2. Ecological Niche Modelling

The ESMs used to predict harbour porpoise habitat suitability and potential distribution showed good performance (CBI 2011 = 0.496; 2012 = 0.961; 2013 = 0.939; 2014 = 0.926; 2015 = 0.815) (Figure S2) although the model built for 2011 was based on a low number of observations and showed lower prediction capacity compared to the other years. Considering this, the 2011 model prediction should be interpreted with caution. Nevertheless, the estimates along the study period are still ecologically relevant.

Considering the variables’ relative contribution to explaining the annual porpoise occurrence probability, the mean chlorophyll concentration (Chl-a) was the most important variable in 2011, 2013, and 2014, whereas bathymetry and SST were the most important variables in 2012 and in 2015, respectively (Table 4).

#### Response Curves

**Figure 4:**
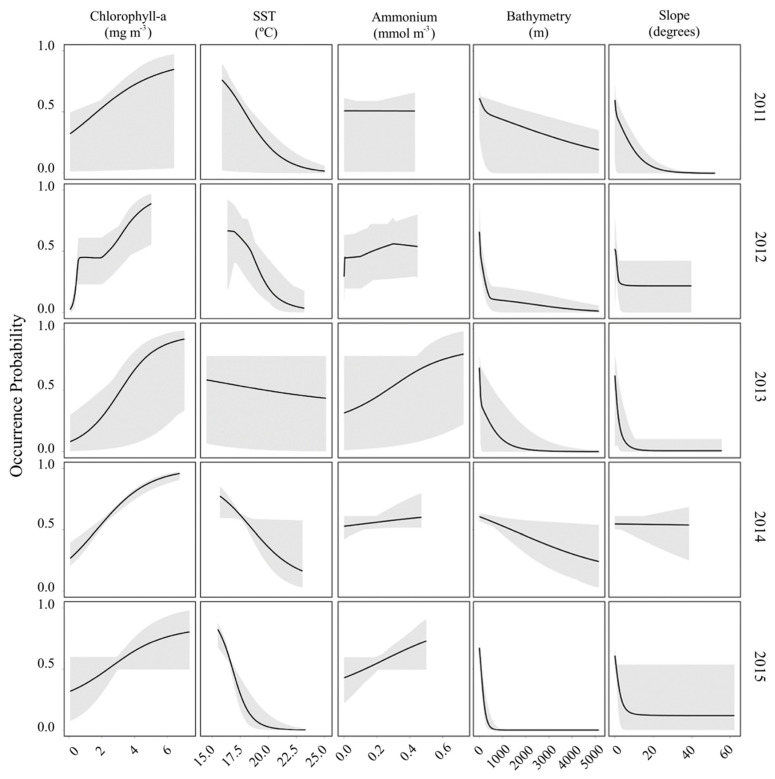
Harbour porpoise occurrence probability response curves in relation to the EGVs included in the ESMs in the study area and extent area during September 2011, 2012, 2014, and 2015 and October 2013. Mean (black lines) and minimum and maximum values (grey areas) of 10 replicates are shown.

The response curves of the ESMs show a marked increase in the environmental suitability with increasing levels of Chl-a in the seawater. The higher HSI values occurred at Chl-a concentrations above 4 mg m^−3^ (Figure 4) in all years. 

Concerning the mean SST, the harbour porpoise occurrence probability generally decreases with increasing SST (Figure 4). However, in 2013 the response curve presents a less pronounced decrease than in the remaining years. In fact, other variables such as Chl-a, bathymetry, and slope present higher contributions to explaining the annual porpoise potential distribution in 2013.

Regarding the mean ammonium concentration, the environmental suitability for the harbour porpoise occurrence shows a general increase in 2013 and 2015 with increasing ammonium levels, although response curves show only a slight or no increase in 2011, 2012, and 2014 (Figure 4). Considering the relative contribution of the selected variables, the ammonium concentration was never identified as the most important variable explaining the annual porpoise occurrence probability.

With respect to the static variables, the bathymetry showed more favorable conditions for the occurrence of the harbour porpoise at shallow waters followed by an estimated environmental suitability decrease with increasing depth. Considering slope, response curves indicate that the most suitable conditions for the harbour porpoise occurred in flat or smooth slope areas compared with areas with higher slopes (Figure 4). However, these results on bathymetry and slope do not hold in 2014 when bathymetry and slope were the least relevant variables to explaining the respective model (Table 4). Instead, in 2014 Chl-a and SST were selected as the most relevant variables.

**Figure 5:**
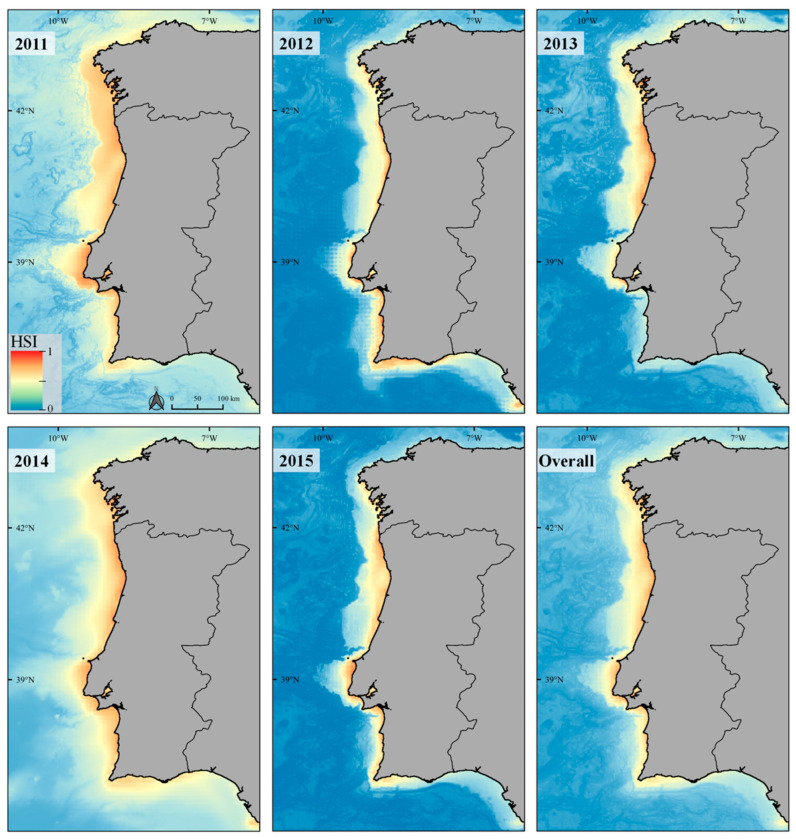
Annual and mean (overall) habitat suitability maps (considering the HSI) for the Harbour porpoise in the study area and the extent of the extrapolation area.

Even though the level of habitat suitability for the harbour porpoise varies annually (Figure 5), in the overall HSI map for the 5 studied years (see overall CV map in Figure S4), the most suitable conditions throughout the Portuguese coastal waters were found in the northern coast. The predictions for the Galician coast (northwestern Spain) also identify areas close to the coast with higher habitat suitability for the harbour porpoise, in particular on the western coast of Galicia, where lower SST values and higher Chl-a concentrations are registered (Figure 5).

## 4. Discussion

### 4.1. Abundance and Density Analysis

In SCANS III (2016) [15], the porpoise abundance estimate for the western Iberian coast was 2715 individuals, and in 2015 the abundance estimate for the Portuguese coast alone was 2254 individuals. Our study reveals that most of the Iberian harbour porpoise population (over ¾ of the population estimated in SCANS III [15]) occupies Portuguese coastal waters. Additionally, the overall habitat suitability is particularly higher in the northern area of the Portuguese coast. Our overall estimates (2011–2015) are well in line with density and abundance values obtained for the AB block in SCANS III [15], corresponding to the western Iberian coast (see Table 5, 0.090 vs. 0.102 ind/km^2^ and 2254 vs. 2715 individuals). However, when considering AA, AB, and AC blocks in SCANS III (corresponding to the harbour porpoise ICES assessment unit) the much larger area leads to a drastically lower density value (0.04 ind/km^2^) confirming the importance of the western Iberian coast to the Iberian porpoise population. This is in agreement with the relatively large number of porpoise strandings recorded over the last 20 years in the western Iberian coast, particularly in the Portuguese northern coast, which concentrates 60% of the total Portuguese and Galician harbour porpoise registered strandings [56].

The large-scale European SCANS II and III surveys [11,15] covered larger areas during summer, and different platforms (ship vs airplane, respectively) were used. However, the sampling area was divided by survey blocks and similar sampling methodologies were used (line transect distance sampling). The 2005 SCANS survey block W covered the coastal area of southwestern France and the Atlantic coast of Spain and Portugal (138,639 km^2^), where an abundance of 2357 porpoises was estimated along with a very low density (0.017 ind/km^2^) (Table 5, [11]). However, these values corresponding to 2005 must be carefully considered given the corresponding large coefficient of variation (92%), probably due to a low number of sightings over a large survey area. Furthermore, the southwestern coast of France in block W might have included harbour porpoise individuals from the admixture zone (between the northeast Atlantic and Iberian porpoise populations), described in the Bay of Biscay [3]. We suggest that the Iberian harbour porpoise population parameters estimated for 2005 in block W should not be used as a baseline for comparisons with the more recent estimates obtained in the present study and in SCANS III [15].

In the present study, the highest annual harbour porpoise abundance and density estimates in Portuguese waters occurred in 2012 (2995 individuals, 0.120 ind/km^2^) and 2013 (3207 individuals, 0.128 ind/km^2^). These estimates were also higher than those obtained for the western Iberian coast in the SCANS III survey (2016) (block AB surveyed in July, [15]). During the 5-year study, despite the relatively high annual coefficients of variation (34.95% < CV < 50.70%), strong population fluctuations were detected between consecutive years. Such annual fluctuations in porpoise abundance estimates in Portugal could be related with the variation in the annual number of dead stranded porpoises. In particular, the lowest population density and abundance estimates occurred in 2011 and 2014 when the highest number of porpoise strandings—38 and 50 individuals, respectively—were registered in Portugal considering the period between 2000 and 2018 [56]. In fact, the population density and abundance in 2014 were approximately half the values obtained in the previous year (see Table 2). It is noteworthy that the 2011 estimated abundance was associated with a relatively high CV (50.70%), so any further inferences about 2011 must be carefully considered. Despite the 23% population increase in the following year (2015), followed by another 24.5% increase in 2016 considering the AB block in SCANS III [15], the density and abundance values remained lower than values estimated in 2012 and 2013, indicating that recruitment did not compensate for mortality (and possibly migration). Since the same porpoise population also inhabits the north of Spain, estimates for the Galician portion of the population (Spanish region bordering Portuguese waters) could have helped to clarify the variation in annual population estimates in Portugal, which could have shown signs of migration or a real decrease in abundance estimates. In 2015, an aerial survey in the Galician coast (Spain) revealed considerably lower harbour porpoise densities (0.014 ind/km^2^) [57,58] than those obtained in the present work. However, considering the low number of sightings, the authors emphasize that the estimate should be considered only a baseline for future evaluations. More recently (2020), a small airplane campaign (976 km) in the southwestern Galician coast registered only one porpoise sighting (two animals) out of 93 cetacean sightings (project VIRADA, [59]). In fact, further Iberian porpoise population estimates in Portugal and Galicia (northern Spain) are needed not only to estimate population trends but also to assess possible seasonal changes in habitat use given the strong seasonal upwelling in this area (see below).

The expected harbour porpoise group size in the present study (Table 2) is in line with the mean group size in other assessments [11,15,60,61], which tends to be rather small, e.g., [62]. However, in the south of Portugal (Algarve sector) the expected group size was relatively higher (Table 3), as previously described from opportunity platform observations in this region (mean group size = 3 individuals, [61]). However, the reason for larger mean group sizes in the South sector remains unclear. Several arguments have been used to explain cetacean group sizes, including SSTs [63,64], prey availability and type of diet [65], anti-predator or social behavior [66], and even collaborative hunting as recently described for porpoises in Denmark [67].

Even though the Iberian porpoise population was thought to be isolated from other porpoises [3,68], there is evidence of porpoise migration to the Iberian area from southern latitudes (Mauritania or an unknown population occurring between the Portuguese south coast and Mauritania) [1], emphasizing the need for studies on African porpoise genetics and population parameters.

When comparing sectors in the present study (North, Centre, Alentejo, and Algarve), the highest estimated porpoise density was registered in the North (0.140 ind/km^2^, CV = 42.43%) and Centre sectors (0.110 ind/km^2^, CV = 39.75%) (Table 3). The SCI Maceda—Praia da Vieira (for which the conservation objective is harbour porpoise protection, [7]) is located in the highest porpoise density sector in the country. Until the present date, no conservation measures were applied whether in or outside the defined SCI. Given the population characteristics (considering genetics and abundance), a proper Iberian population action plan, including adequate measures and new protection areas, is urgently needed in the Iberian Peninsula.

### 4.2. Ecological Niche Model

In the study area, the models predicted the most suitable conditions for harbour porpoise occurrence were found predominantly in coastal and shallow waters, as already described in other studies considering different populations of porpoise, e.g., [69,70,71]. Coastal shallow waters in the Iberian western coast are strongly influenced by upwelling [72], which explains Chl-a concentration as one of the most important predicting variables, also in accordance with other studies focused on cetaceans and particularly on the harbour porpoise [73,74,75,76]. Chl-a concentration is widely accepted as a proxy for areas of enhanced biological production (see [77]), where primary consumers tend to aggregate [78] contributing to defining prey distribution (fish, in this case), eventually leading to important feeding grounds for marine predators [79].

However, in 2012 the occurrence probability variation with increasing Chl-a concentrations presents a different pattern when compared to the remaining study years: an initial sharp increase at relatively low Chl-a concentrations (<1 mg m^−3^) followed by a plateau, and a new steady, less pronounced increase in porpoise occurrence probability at higher Chl-a concentrations (>2 mg m^−3^), similar to the other years. One of the highest harbour porpoise abundance estimates was obtained in 2012 when the highest habitat suitability was detected in a very narrow portion of the central and northern Portuguese coast and in the southwestern coast (south of Sines). Oscillations of the Iberian upwelling system may contribute to porpoise annual (and even seasonal) abundance and density variations. It is possible that upwelling phenomena become more irregular in duration and strength towards the end of summer [80]. Apart from Chl-a concentrations, SST was also an important variable in guiding harbour porpoise distribution, as identified in other study areas [69,74]. The SST effect is particularly important in 2013 when the highest porpoise abundance estimates were obtained. The lower SSTs in the Centre and North sectors in relation to the higher SSTs in the (south) Alentejo and Algarve sectors led to the widest annual SST range across the study area (14.5–25.2 °C) in 2013 and probably explains the concentration of the Iberian porpoise population in the Centre and North sectors during that year.

In addition, it is concerning that in 2013 (corresponding to the highest annual abundance estimate) a low habitat suitability was estimated in the Alentejo and the Algarve sectors. As mentioned earlier, high Chl-a concentrations and lower SST values in the Centre and North sectors contributed to the concentration of the potential porpoise distribution in these coastal areas. The fact that the 2013 survey was carried out in early October, whereas in the remaining years surveys were carried out in September, may account for some of the variation. However, the highest annual abundance estimate (in 2013) was followed by the highest annual number of stranded porpoises in Portugal [56], which is most likely associated with the abundance reduction (in 2014) by nearly 50% in comparison to the previous year. This abrupt decrease in harbour porpoise abundance (Table 2) appears to be associated with an SST increase and a decrease in Chl-a and ammonium concentrations in the north/centre of Portugal (Figure S3), which in turn may be associated to a decreased prey availability. Marine fish landings did decrease in 2014 in relation to previous years, except for the European seabass (*Dicentrachus labrax*) [81,82,83,84,85]. This high commercial-value species is one of the main target species for illegal, unreported, and unregulated (IUU) fisheries (nets operating very near the coastline), which could indicate that a higher than usual fishing effort was used in areas where porpoises concentrate. Increased surveillance of coastal gill/trammel nets operating illegally near the coast (mainly distance from coast < 0.25 nm, some drift nets, etc.) could be a promising deterrent measure for porpoise bycatch in the study area.

The habitat suitability maps for the harbour porpoise in the study area (including Galicia, Spain, a part of the extent area) emphasize the importance of coastal areas between Nazaré (central coast, Portugal) and the western coast of Galicia (Spain). Even though an MPA dedicated to the protection of porpoises was recently designated in the central coast of Portugal (SCI Maceda—Praia da Viera), there are no other protected areas with representative marine areas in northern Portugal or western Galicia. However, the definition of a critical area for porpoises was proposed in 2018 in western Galicia [86], and porpoises are now officially declared as Critically Endangered (En Peligro de Extinción) in Spain [87].

Results indicate that a transnational marine protected area could be important for the conservation of the Iberian harbour porpoise [88]. Other important areas are visible in coastal areas south of Cape Carvoeiro (Peniche), south of Sines and around Cape São Vicente, although their importance varies between years. Nonetheless, marine protected areas are not useful to the conservation of the Iberian harbour porpoise unless monitoring and threat mitigation measures are put in place (see [8]).

The seasonal and annual variability of the Iberian upwelling system emphasizes the importance of seasonal porpoise surveys in the future, to fully understand the spatial and temporal porpoise habitat use in the Iberian Peninsula. Within areas with overall higher habitat suitability, porpoises may use different portions of the available habitat according to varying environmental conditions and resources. Seasonal changes in the distribution of harbour porpoises have been suggested in Europe [89,90,91] as well as in previous Portuguese land-based observations [92,93]. Therefore, seasonal movements of this porpoise population within Iberian waters may occur, and systematic monitoring is required to detect them.

Considering the harbour porpoises’ need for a constant energy intake [94,95], it is possible that climate/environmental-driven changes may continue to affect (foraging) distributions as hypothesized for the redistribution of harbour porpoises and separation into different populations and subspecies [3]. On a much smaller time scale, harbour porpoise diet analyses in Portugal have revealed that the most consumed species varied over time [96,97] indicating that Iberian porpoises can adapt to the available prey species, as described in other studies [98].

The overall HSI map (for the period between 2011 and 2015) suggests a higher habitat suitability in central-northern Portugal (Figure 5), corroborating the importance of the SCI Maceda—Praia da Vieira (PTCON0063) legally approved in 2019. Despite being a highly mobile species, this SCI should encompass (during the study period) at least 32% of the harbour porpoise population estimated for the Portuguese mainland EEZ. Until 2021, the majority of annual Iberian porpoise strandings continues to occur on the northern coast of Portugal [56].

### 4.3. Limitations of the Study

Accurate abundance and density estimates depend on several factors affecting field surveys (flight and environmental conditions affect perception whereas species size, morphology, and behavior affect availability or detectability bias). In this study, the bias levels can be considered constant since the platform, observers, and field protocols were the same in all studies [13]. Furthermore, the use of the most recent correction factor (SCANS III g(0) = 0.364) [15] for the target species allows for more accurate results since the probability of detecting the animals at distance zero from the transect line are underestimated by an unknown magnitude if the estimates were not corrected for availability and perception bias [99]. This factor was estimated during the SCANS III survey under conditions very similar to those in the present study. Nonetheless, this correction factor could be improved when enough observation effort, sightings, and circle-back procedures are available for airplane campaigns in Portuguese waters in the future. Although other methods are possible (using two simultaneous airplanes or double platforms in one airplane), circle-back procedures allow for an ESW estimate, weighted for the proportion of animals observed on the transect line, and they also take into account the assumed decline in that proportion with distance from the transect line [100]. Such methodology is important in aerial surveys because of the limited time that any animal is available for detection [11]. Using the previously available correction factor from SCANS II (g(0) = 0.45) [11], the porpoise abundance (and density) estimates for the study area were relatively lower (overall abundance 1523 porpoises, see Table 6). A trend with less-accurate g(0) can still be a helpful indication to emphasize the need for conservation measures in more urgent scenarios, such as the Iberian harbour porpoise [7]. Nevertheless, improved correction factors are extremely important since abundance estimates are then used to estimate population trends and potential biological removal values, which dictate the form and urgency of species conservation measures.

A further limitation in this study was the yearly small sample size used for the potential distribution analysis. To decrease the predictive uncertainty, the assessment of harbour porpoise habitat suitability was performed using ESMs [17], which show a high performance with small sample sizes, such as the case of rare and threatened species, or elusive species that are hard to detect [16,43]. In addition, MaxEnt was used as the ESMs algorithm since it is considered very efficient with irregular data and with small sample size data [44,45]. The 2011 ESM showed lower prediction capacity (CBI = 0.496) compared to the other years, and results should be interpreted with caution. Nevertheless, the estimates for the study area are still ecologically plausible.

## 5. Conclusions

The porpoise densities estimated in the present study in Portugal between 2011 and 2015 (0.090 ind/km^2^, CV = 21.99%) and one year later for the western Iberian coast (0.102 ind/km^2^, CV = 30.8%) [15] are even lower than the recently estimated harbour porpoise populations of the Black Sea subspecies (0.339 ind/km^2^, CV = 9.91% for a design-based analysis) [61] currently classified as Endangered in the IUCN red list [101]. Likewise, a large discrepancy is verified between the most recent density values of harbour porpoise inhabiting in Portugal and in the Baltic Sea (1.15 ind/km^−2^, CV = 28.5%) [15]. Highlighting that the porpoise subpopulation inhabiting the Baltic Sea waters (thought to be restricted to the Baltic and Kattegat sea) is currently classified as Critically Endangered in the IUCN red list [102].

The annual fluctuations in harbour porpoise abundances in Portugal revealed an abrupt decrease in 2014. Whether this abundance decrease in Portugal was due to mortality or to displacement to other areas (nearshore–offshore movements are unlikely but migration to other latitudes outside the study area is plausible) remains unclear. Larger-scale coordinated and periodic surveys covering at least the entire Iberian population are needed to better understand the annual abundance fluctuations detected in the present study.

This study emphasizes the importance of transboundary conservation efforts involving Spain and Portugal, including further porpoise population assessments and the need for on-site threat mitigation measures supported by legal frameworks involving the development of human activities (fisheries, including IUU fisheries and other emergent blue economy sectors). Despite the efforts to legally approve an SCI dedicated to the protection of harbour porpoises in Portugal under the Habitats Directive framework, no further measures were approved to halt the porpoise population decrease.

## Figures and Tables

**Table 1:** Eco-geographical variables (EGVs, units, resolution, and source) extracted from online repositories and incorporated in the preliminary analysis.

	EGVs	Units	Original Resolution	Source
Static variables	Bathymetry	Meters	30 arc-sec (~1 km)	[36] http://marspec.org/, accessed on 18 June 2021 Distance to coast and slope: Derived from bathymetry
	Distance to coast			
	Slope	Degrees	30 arc-sec (~1 km)	
Dynamic variables	Dissolved oxygen (O_2_)	mmol m^−3^	0.083° (~8 km) (Monthly average, lag-1)	Copernicus Atlantic-Iberian Biscay Irish-Ocean Physics Reanalysis (CMEMS) https://resources.marine.copernicus.eu/product-detail/IBI_MULTIYEAR_BGC_005_003/INFORMATION, accessed on 18 June 2021
	Nitrate (NO_3_)			
	Ammonium (NH_4_^+^)			
	Phosphate (PO_4_)			
	Silicate (Si)			
	Iron (Fe)			
	Chlorophyll concentration (Chl-a)	mg m^−3^		
	pH	-		
	Salinity	Psu		Copernicus Atlantic-Iberian Biscay Irish-Ocean Physics Reanalysis (CMEMS) https://resources.marine.copernicus.eu/product-detail/IBI_MULTIYEAR_PHY_005_002/INFORMATION, accessed on 18 June 2021
	Sea Surface Temperature (SST)	°C		

**Table 2:** Harbour porpoise abundance and density estimates, using g(0) = 0.364 [15], in Portugal between 2011 and 2015. Survey effort (number of transects and length), no. of harbour porpoise sightings and individuals, and expected group size. CV, coefficient of variation (%). CI, confidence interval.

Survey Year	Transects	Distance (km)	Sightings	Individuals	Expected Group Size (CV)	Density (ind/km^2^) (95% CI)	Abundance (95% CI)	CV
						g(0) = 0.364		
2011	39	1445.3	6	8	1.32 (13.72)	0.048 (0.018–0.125)	1196 (456–3135)	50.70
2012	40	1482.4	16	23	1.28 (10.61)	0.120 (0.061–0.236)	2995 (1516–5917)	34.95
2013	40	1482.4	16	25	1.42 (21.11)	0.128 (0.061–0.268)	3207 (1531–6718)	38.14
2014	37	1408.3	8	11	1.32 (16.55)	0.066 (0.029–0.152)	1653 (717–3809)	43.27
2015	39	1482.4	10	15	1.44 (15.79)	0.086 (0.037–0.199)	2147 (923–4997)	43.86
Total	195	8635.0	56	83	1.39 (7.80)	0.090 (0.051–0.158)	2254 (1287–3949)	21.99

**Table 3:** Harbour porpoise abundance and density estimates, using g(0) = 0.364 [15], in the different sectors in Portugal during the study period. Survey effort (number of transects and length, in km), no. of harbour porpoise sightings and individuals, and expected group size. Coefficient of variation (CV, %) and confidence interval (CI) were estimated considering the 5-year sampling period.

Survey Sector	Transects	Distance (km)	Sightings	Individuals	Expected Group Size (CV)	Density (ind/km^2^) (95% CI)	Abundance (95% CI)	CV
						g(0) = 0.364		
North	58	6710.99	27	34	1.26 (7.44)	0.140 (0.061–0.318)	935 (409–2139)	43.42
Centre	56	9145.15	17	26	1.52 (8.16)	0.110 (0.051–0.237)	1010 (470–2168)	39.75
Alentejo	32	3424.86	4	5	1.14 (23.10)	0.033 (0.012–0.092)	113 (40–315)	53.90
Algarve	49	5771.52	8	17	1.90 (24.79)	0.071 (0.026–0.197)	412 (150–1136)	55.99

**Table 4:** EGVs relative contributions (%) to explaining the annual porpoise occurrence probability in each annual ESM. Highest annual contributions are shown in bold.

EGVs	2011	2012	2013	2014	2015
Bathymetry	20.599	**20.691**	20.562	19.093	20.188
Chl-a	**20.619**	20.663	**21.313**	**21.786**	20.452
Ammonium	19.835	19.385	18.970	19.556	18.959
SST	19.716	20.570	19.129	20.046	**21.335**
Slope	19.231	18.691	20.026	19.519	19.066

**Table 5:** Harbour porpoise abundance and density values (CV, confidence intervals) obtained in SCANS II [11] and SCANS III blocks that include the Iberian Peninsula [15], and in the present study.

	Survey Block	Location	Platform	Survey Years	Area (km^2^)	Abundance (*n*)	Density (*n*/km^2^)	CV (%)
SCANS II	W	SW France and Atlantic Iberian Peninsula (Portugal and Spain)	ship	2005	138,639.00	2357	0.017	92
Present study		Portuguese Coast	airplane	2011–2015	25,052.52	2254	0.090	21.99
SCANS III	AB	Western Iberian coast (Portugal and Galicia, Spain)	airplane	2016	26,668.00	2715	0.102	30.8
SCANS III	AA + AB + AC	Atlantic Iberian Peninsula ^1^ (Portugal and Spain)	airplane	2016	72,863.00	2898	0.04	32.00

^1^ considered as equivalent to the harbour porpoise ICES assessment unit.

**Table 6:** Harbour porpoise density (ind/km^2^) and abundance estimates in Portugal between 2011 and 2015, using g(0) = 0.45 (see Methods: defined for good observation conditions in SCANS II, [11]). CI, confidence interval, CV, coefficient of variation (%).

Survey Year	Density (95% CI)	Abundance (95% CI)	CV
	g(0) = 0.45		
2011	0.039 (0.016–0.100)	991 (391–2515)	48.90
2012	0.096 (0.053–0.174)	2416 (1338–4363)	30.22
2013	0.120 (0.059–0.246)	3020 (1482–6157)	36.78
2014	0.057 (0.026–0.125)	1437 (658–3135)	40.36
2015	0.074 (0.034–0.162)	1859 (853–4051)	40.32
Total	0.061 (0.029–0.126)	1523 (733–3163)	31.19

## Data Availability

The data presented in this study are available on request from the corresponding author. The data are not publicly available because they were obtained under particular data sharing protocols, and they are still in use by the corresponding author.

## Supplementary Materials

The following supporting information can be downloaded at: https://www.mdpi.com/article/10.3390/ani12151935/s1, Figure S1: Pearson’s correlations (tree diagrams) for EGVs included in the harbour porpoise habitat suitability models. Red boxes indicate highly correlated variables (>0.7). SST—Sea Surface Temperature; Figure S2: Violin plot representing the distribution of the Continuous Boyce Index (CBI) in different years. The score of each single model (grey dot), average score for single models (red dot), and the score for each of the ensemble models (blue dot) are also shown; Figure S3: Dynamic EGVs incorporated in the final model for harbour porpoise in Western Galician and Portuguese continental waters using the Maxent algorithm; Figure S4: Coefficient of variation of the probabilities using the annual models.
