# Supplementary material for: Harbour Porpoise Abundance in Portugal over a 5-Year Period and Estimates of Potential Distribution
Source: Animals (Basel). 2022 Jul 29;12(15):1935. doi: 10.3390/ani12151935 (PMC9367303; doi:10.3390/ani12151935)
Supplement: Supplementary file 1 [file animals-12-01935-s001.zip › animals-1766381-supplementary.pdf]

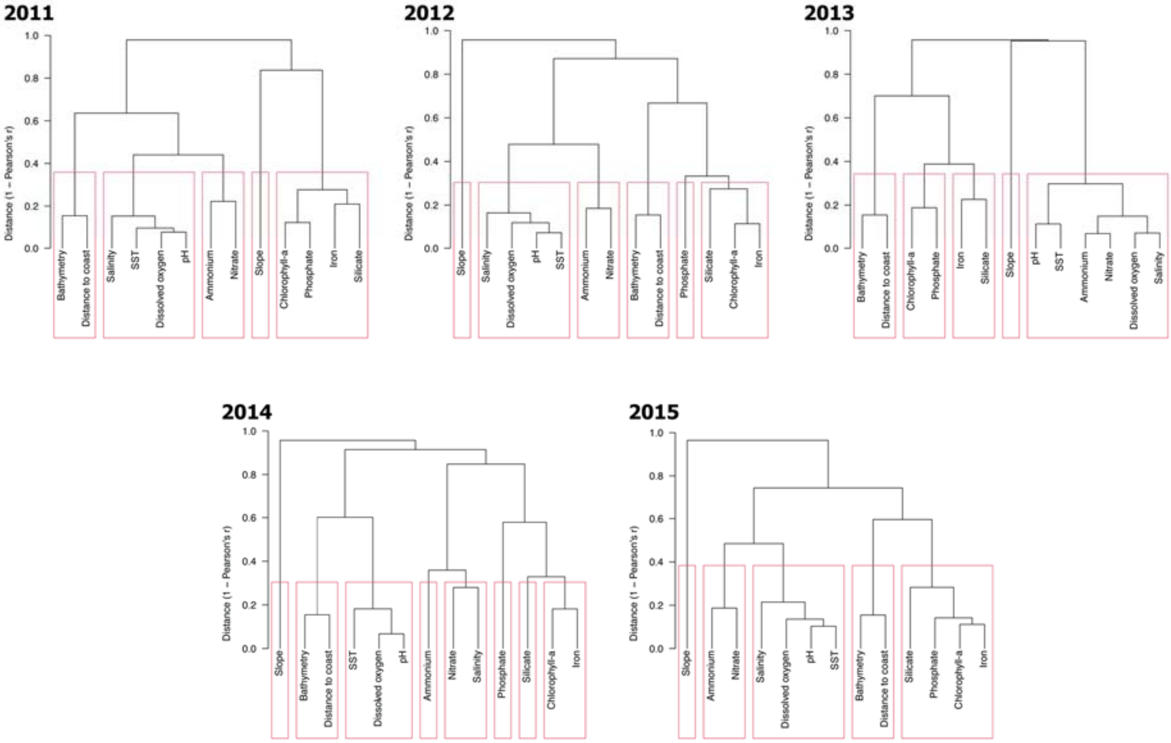

Figure S1. Pearson's correlations (tree diagrams) for EGVs included in the harbour porpoise habitat suitability models. Red boxes indicate highly correlated variables (>0.7). SST—Sea Surface Temperature.

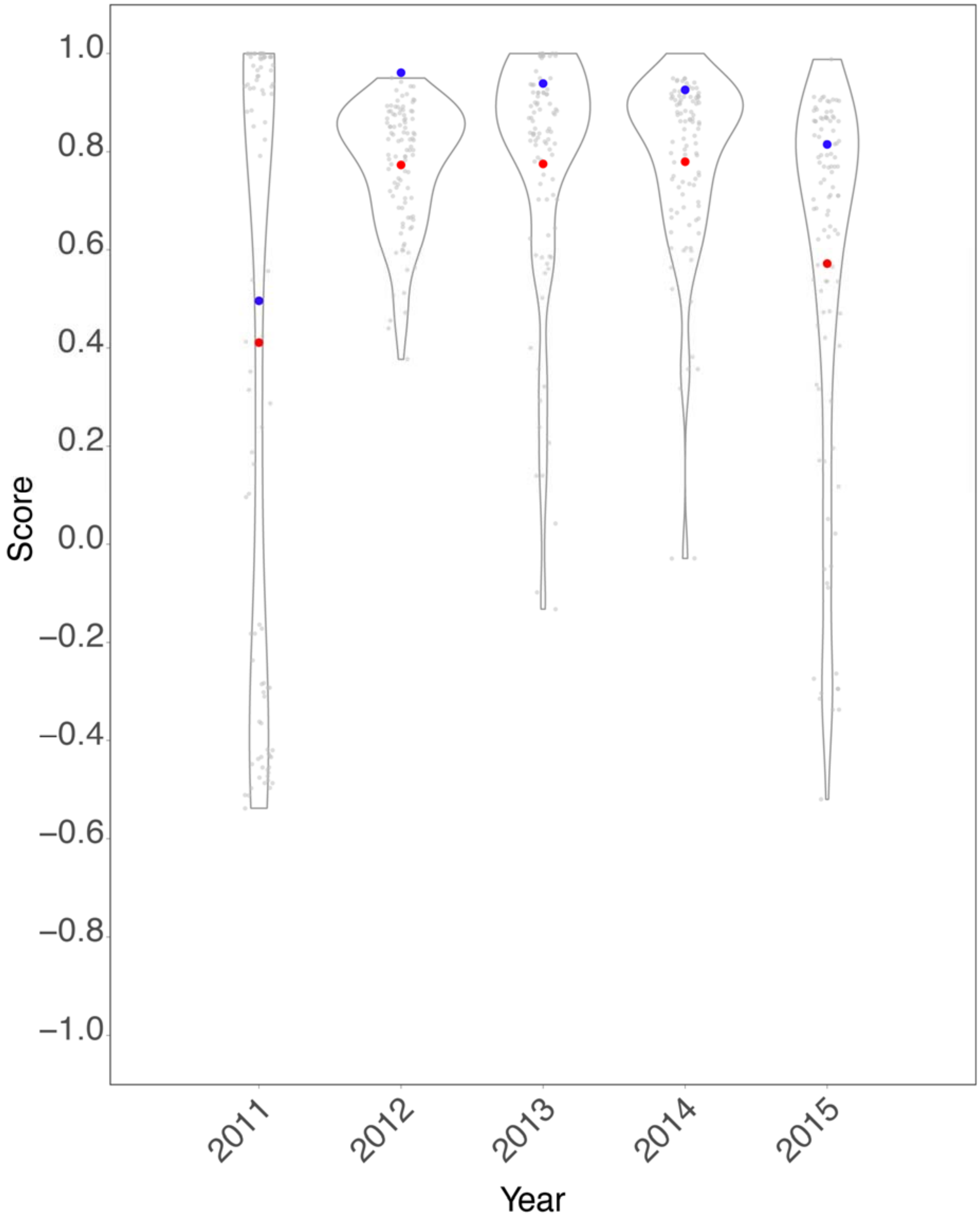

Figure S2. Violin plot representing the distribution of the Continuous Boyce Index (CBI) in different years. The score of each single model (grey dot), average score for single models (red dot), and the score for each of the ensemble models (blue dot) are also shown.

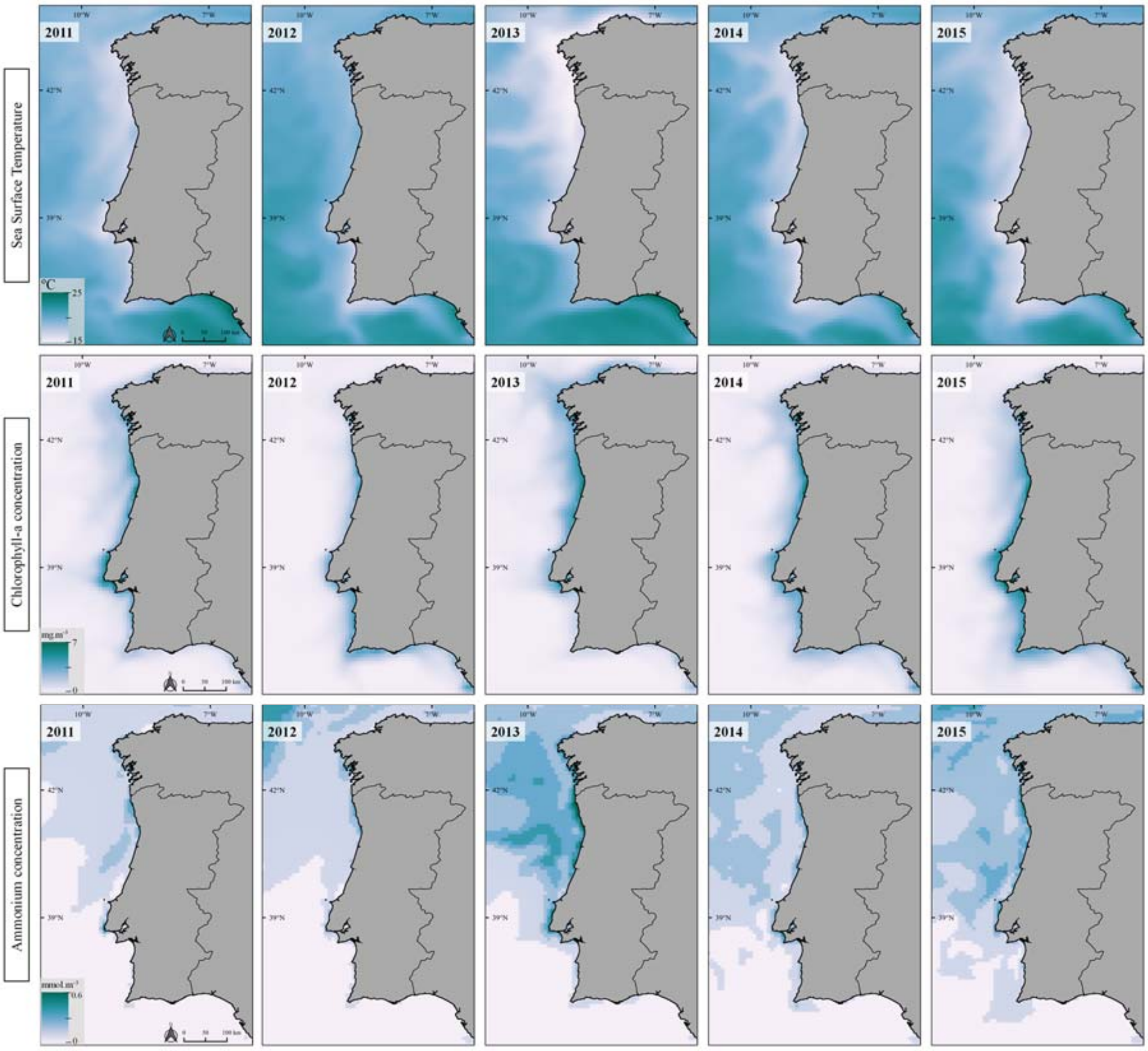

Figure S3. Dynamic EGVs incorporated in the final model for harbour porpoise in Western Galician and Portuguese continental waters using the Maxent algorithm.

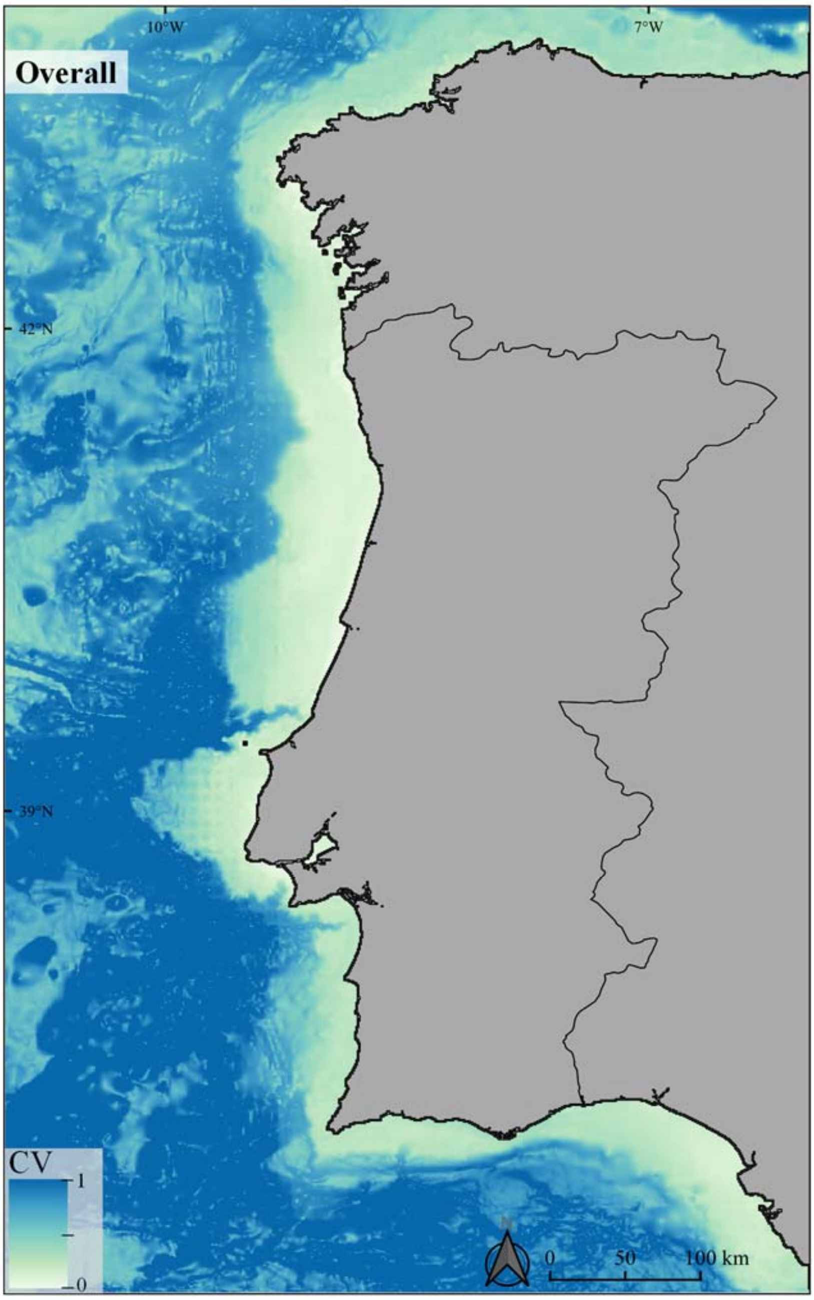

Figure S4. Coefficient of variation of the probabilities using the annual models.
